# Influence of Cement Type on the Performance and Durability of Cement Paste and Concrete with Wastewater

**DOI:** 10.3390/ma19020435

**Published:** 2026-01-22

**Authors:** Eirini-Chrysanthi Tsardaka, Eleftherios K. Anastasiou, Aikaterina Karanafti, Juan Antonio Ferriz-Papi, Jan Valentin, Theodoros Theodosiou

**Affiliations:** 1 Department of Civil Engineering, Aristotle University of Thessaloniki, 54124 Thessaloniki, Greece; extsardaka@gmail.com (E.-C.T.); akaranaf@civil.auth.gr (A.K.); tgt@civil.auth.gr (T.T.); 2 School of Science, Engineering & Environment, University of Salford, Manchester M5 4WT, UK; j.a.ferriz-papi@salford.ac.uk; 3 Department of Road Structures, Czech Technical University in Prague, 166 36 Prague, Czech Republic; jan.valentin@fsv.cvut.cz

**Keywords:** cement hydration, blended-type cements, concrete truck wastewater, carbonation resistance, chloride penetration

## Abstract

Recycling wastewater from washing concrete trucks in concrete production addresses both economic and sustainability needs. In the present article, wastewater from washing concrete trucks was added to cement pastes made with two different types of cement for comparison. OPC type CEM I 42.5 was compared to pozzolanic cement type CEM IV/B (P-W) 32.5 in terms of hydration behavior and compressive strength development. The hydration of ordinary Portland cement (CEM I 42.5) was accelerated, while the hydration of pozzolanic cement (CEM IV 32.5) showed a relatively lower total normalized heat. Cement pastes were produced from both cement types, and compressive strength, thermal analysis, and setting time tests were performed for their characterization. The early-age kinetics and compressive strength development of CEM I 42.5 pastes indicate that hydration with wastewater leads to a slight increase in compressive strength. Test concrete prepared with pozzolanic cement (CEM IV 32.5) exhibited increased capillary voids, which contributed to less favorable mechanical and durability performance. Compared to the reference concrete, compressive strength was reduced by 7% at 28 days. Wastewater utilization increased the initial absorption rate by approximately 20%, but the calculated chloride content at the exposed concrete surface decreased after the addition of wastewater compared to the control mix. The carbonation depth of concrete with wastewater increased by 1–2 mm, with an uneven penetration zone, but the compressive strength after carbonation increased. Overall, the type of cement used appears to significantly influence the performance of concrete prepared with wastewater. For wastewater collected from sedimentation tanks, replacing fresh water at a 100% rate and using it with pozzolanic cement to produce concrete, it seems that the mechanical properties and durability are only slightly affected.

## 1. Introduction

The annual global production of concrete is expected to exceed 14 billion m^3^ in the coming years [1]. During concrete production, large amounts of water are used for washing aggregates, cutting stone and marble, washing concrete trucks, and as mixing water. Wastewater from concrete truck washing is generated in significant quantities in the cement and concrete industry, estimated at around 90 kg of water per m^3^ of concrete from truck washing, although the actual values may range from 13 to 500 kg/m^3^ depending on production practices, as noted by Mack-Vergara and John [2]. Untreated wastewater discharge from the concrete industry poses a significant threat to the environment and public health because it contains heavy metals and has a high pH [3]. This wastewater can cause contamination and ecological imbalances, affecting both ecosystems and human health. Mack-Vergara [4] identifies four main environmental impacts from water use in concrete production: water consumption, acidification, eutrophication, and toxicity. Therefore, recycling wastewater in the construction industry would be valuable for health, environmental, and economic reasons.

Directly recycling concrete truck wastewater as mixing water in fresh concrete production may cause defects in the final product, primarily reduced workability and increased setting time [5]. However, it is possible to use concrete truck wastewater as mixing water for fresh concrete by applying various mechanical and chemical treatments [6]. One of the most common mechanical methods is deposition in tanks [7]. During deposition, sedimentation occurs as particles settle at the bottom of the tank, leaving water at the top. This water, which contains water-soluble content and a few particles, is then transferred to a second tank. A series of four consecutive tanks ensures the separation of solid particles and purifies the water from most solids. This type of wastewater has been successfully used in concrete production to replace clean tap water [5,8]. Tap water has a neutral pH (below 8) and contains fewer soluble salts and anions compared to wastewater. As a result, comparing concrete made with wastewater to conventional concrete presents some differences. Numerous reports, thoroughly reviewed in [9,10], note changes in fresh properties such as workability and slump, with authors reporting a reduction in slump due to the addition of wastewater.

The wastewaters tested in the literature exhibited various pH values depending on their source and alkalinity, as reviewed by Varshney et al. [9]. de Matos et al. [5] reported that the high pH of wastewater may affect the surface electrical properties of cement powder, resulting in increased flocculation. Chatveera et al. [11,12] found that using alkaline wastewater from concrete truck wash, with pH values above 11, provided stable hydration strength but reduced durability. Additionally, the alkalinity of the water resulted in a more porous and weaker matrix that was more susceptible to acid attack. Consequently, investigating the effects of alkaline wastewaters on concrete durability is challenging.

Some researchers observed a small increase in compressive strength when using secondary treated wastewater with a neutral pH [13,14], while others reported a slight decrease when using wastewater from concrete washing, which had a higher pH of around 11–12 [5,15,16]. This small variation could be attributed to differences in the chemical composition of the cements or the composition and alkalinity of the wastewaters used. Chen et al. [7] used P-II-52.5 cement and measured a significant increase in compressive strength as the amount of wastewater increased. The authors concluded that the wastewater contained cement, fly ash, and slag particles, which acted as filler material in the concrete structure and increased its density. Vaičiukynienė et al. [17] reached a similar conclusion, attributing the increase in compressive strength to the pozzolanic reaction of the zeolitic by-product used in the mix. In most of the above-mentioned studies, however, Ordinary Portland Cement (OPC) was used for testing cement pastes or concrete with wastewater. Over the past decades, blended cement types have become increasingly preferred over OPC in construction for environmental and financial reasons [18]. Recent investigations have also advanced the understanding of the role of supplementary cementitious materials in concrete [19]. Therefore, it is necessary to fully explore the effects of using wastewater as mixing water in concrete produced with blended cements.

In the present study, the behavior of two different cement types was evaluated to highlight differences in hydration kinetics. Subsequently, CEM IV/B (P-W) 32.5 was used for laboratory concrete production with both tap water and wastewater to determine slump, density, flexural and compressive strength, as well as concrete durability against carbonation and chloride attack. The research project aimed to identify the role of cement type in the properties of concrete produced with 100% recycled wastewater from concrete truck washing.

## 2. Materials and Methods

### 2.1. Preparation of Mixing Water

When concrete trucks return from the field and are washed with tap water, the resulting wastewater is collected in a tank where most particles settle. The excess water then overflows into an adjacent tank. This process is repeated through four consecutive tanks, and the final water used for this experimental program was taken from the fourth tank in the series. Each tank used had a volume of approximately 100 m^3^, the sedimentation process lasted 12 h, and no stirring was applied. The water used for concrete production should comply with the requirements of European Standard EN 1008 [20], making it suitable for concrete mixtures.

### 2.2. Preparation of Cement Pastes

According to EN 197-1 [21], CEM I 42.5 contains 95–100% clinker and 0–5% minor constituents, while CEM IV/B (P-W) 32.5 contains 45–64% clinker, 36–55% natural pozzolan and calcareous fly ash, and 0–5% minor constituents. These two cements used were tested for their density [22], their chemical properties, and their particle size distribution.

Reference and test cement pastes were prepared using tap water and 100% by weight replacement with wastewater, respectively. The cement pastes were evaluated for 28 days, while heat of hydration was recorded during the first 60 h to monitor the hydration process. Setting times were measured to indicate any delay or acceleration in setting and hardening. Thermal analysis was conducted to compare portlandite content at 7 and 28 days. Compressive strength tests at 7 and 28 days assessed the impact of replacing tap water with wastewater. Paste evaluation helped to understand the behavior of the clinker and additives in the presence of wastewater.

The behavior of the two different cement types mixed with tap water and wastewater, respectively, was tested. Pastes prepared with CEM IV 32.5 were designated as CRp, and those prepared with CEM I 42.5 were designated as Rp, both followed by the number 100 when 100% of the water was replaced with wastewater. Cement and water quantities were weighed and mixed under a mechanical stirrer to achieve standard consistence according to EN 196-3 (Vicat penetration 6 ± 2 mm) [23]. Table 1 summarizes the design of the cement pastes with their respective consistence. The pastes were cast into specimens with dimensions of 25 × 25 × 50 mm^3^ and 25 × 25 × 100 mm^3^. After one day, the specimens were demolded and cured at 20 ± 1 °C, then immersed in water until the mechanical properties were tested at respective ages.

### 2.3. Testing of Cement Pastes

The heat of hydration of the cement pastes was measured by isothermal calorimetry, and the compressive strength of the cement pastes was measured at 7 and 28 days, according to EN 196-1 [24], using cubic specimens with dimensions 10 × 10 × 10 cm^3^. After the completion of the compressive strength test, a sample from the center of each specimen was ground for portlandite determination using thermal analysis.

### 2.4. Preparation of Concrete

The proportioning of concrete prepared with CEM IV 32.5 is shown in Table 2. CEM IV 32.5 was chosen for the concrete mixtures because it is one of the most commonly used cement types for construction in Greece. The concrete mix was produced with tap water for specimens designated as CR and was repeated with a 100% by weight replacement of tap water with wastewater for specimens designated as CR100. The slump test was performed immediately after concrete production, following EN 12350-2 [25], and was repeated after 60 min. Apparent specific density was measured according to EN 12350-6 [26].

### 2.5. Testing of Concrete

The use of wastewater in replacement of tap water may affect properties of concrete such as strength, porosity, and durability. The flexural and compressive strengths of concrete were determined at 28 and 90 days using 100 × 100 × 400 mm^3^ prisms and 150 × 150 × 150 mm^3^ cubes, respectively, in accordance with EN 12390-3 [27]. Porosity was assessed indirectly by the water permeability and the capillary absorption tests. The durability of the test concretes was assessed by the accelerated carbonation and chloride diffusion tests, along with analytical tests such as Thermal Analysis and X-Ray Diffraction.

### 2.6. Methods of Investigation

The residual mass of wastewater was determined by heating a quantity of wastewater in an oven at 110 °C. The pH value was measured using a glass electrode, and the density was determined according to the method specified in EN 1008 [10]. The sodium oxide equivalent content (Na_2_O_eq_) was determined using Atomic Absorption Spectroscopy (AAnalyst 400, Perkin Elmer, Shelton, CT, USA). Water-soluble chlorides, nitrates, and sulfates were measured by Ion Chromatography.

X-ray fluorescence (XRF) was used to characterize the cement powders, employing a Tiger S8 from Bruker Instruments, Billerica, MA, USA. Ion chromatography (IC) was used to determine water-soluble chlorides, nitrates, and sulfates in wastewater and cement powders, as well as to measure chlorides in concrete after the chloride attack test. A Dionex ICS-1100 from Thermo Scientific Instruments, Waltham, MA, USA (eluent buffer: 4.5 mM Na_2_CO_3_/1.4 mM NaHCO_3_, flow rate: 1.2 mL/min, detection: suppressed conductivity) was used for IC. Laser particle size distribution (PSD) analysis was performed to determine the granulometry of cement powders using a Mastersizer 2000 (Malvern Instruments, Malvern, UK).

The setting times of standard-consistency cement pastes were determined using an automatic Vicat apparatus, Vicatronic Matest, Treviolo, Italy. The flexural and compressive strength tests of cement pastes and concrete specimens were performed in a 2000 kN universal concrete testing machine (Matest, Treviolo, Italy), according to the relevant standards. The heat of hydration for the pastes was assessed using the TAM Air eight-channel iso-thermal calorimeter, TA Instruments, New Castle, DE, USA, in accordance to the relevant standard [28]. This device enables the continuous monitoring of heat evolution in pastes, with a tested sample volume of 5 mL. During each test, data were recorded continuously for 60 h. Thermal analysis was carried out using a Netzsch STA 449 F5 Jupiter, Selb, Germany, under a nitrogen atmosphere (50 mL per minute) from 50 °C to 1000 °C at a heating rate of 10 °C per minute.

X-Ray diffraction analysis was used for the investigation of NaCl formation with a D2 Phaser 2nd generation, Bruker Instruments. X-Ray diffraction patterns were recorded at Cu Ka (30 kV and 10 mA, λ = 1.540 A) from 2° θ to 75° θ, with step 0.02° θ and time per step 0.4 s. EVA V5.0 software (Bruker, Billerica, MA, USA) and COD database (Crystallography Open Database) were used for the identification of XRD functions and the diagrams.

The depth of water penetration under pressure in concrete was determined according to EN 12390-8 [29] using 100 × 100 × 100 mm^3^ cubic specimens, and the water penetration depth was measured in millimeters. The rate of water absorption by hydraulic cement concretes was measured according to ASTM C1585-04 [30].

The carbonation resistance of concrete was determined using the accelerated carbonation method according to EN 12390-12 [31]. Prisms measuring 100 × 100 × 400 mm^3^ were cured in a climatic chamber with 3% CO_2_, as indicated by the standard, and carbonation was evaluated with the phenolphthalein test after 7, 28, and 70 days in the chamber. Additionally, at 70 days, the calcium carbonate content was determined by Thermal Analysis in the temperature range of 600 °C to 800 °C. The compressive strength of specimens cured in CO_2_ was measured at the same ages to compare with the compressive strength of specimens cured in humid conditions. After compression, a small sample was collected from a depth of 1 cm from the surface, and the cement paste was carefully separated from the aggregates. This sample was used to determine the calcium carbonate content of the systems by Thermal Analysis.

The chloride resistance of concrete by unidirectional diffusion was determined according to EN 12390-11 [32]. Cubic specimens measuring 100 × 100 × 100 mm^3^ were saturated with water and treated with calcium hydroxide solution for 18 h, as specified by the standard, and then immersed in a 3% by mass sodium chloride (NaCl) solution for 90 days. After 90 days, the cubes were removed from the NaCl solution, and material was drilled from the specimens to depths of up to 40 mm from the surface. The chloride content (water-soluble chlorides) at each specified depth was determined using ion chromatography.

## 3. Results and Discussion

### 3.1. Characterization of Materials

The wastewater was characterized before the experimental phase, and its tested characteristics are shown in Table 3. According to the results, the characterized water meets the requirements of EN 1008 and can be used in cement mixtures and concrete production.

The characterization of CEM IV/B (P-W) 32.5 (hereafter referred to as CEM IV 32.5) and CEM I 42.5 is presented in Table 4.

**Figure 1:**
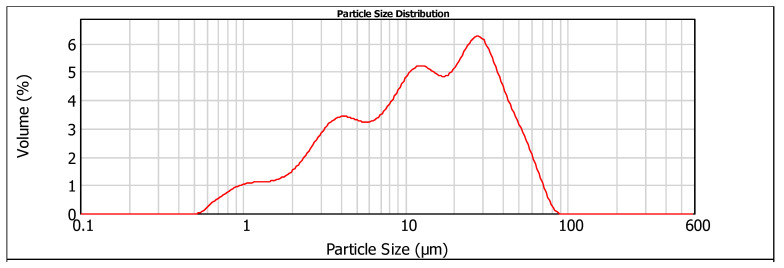
Particle size distribution analysis of CEM I 42.5.

**Figure 2:**
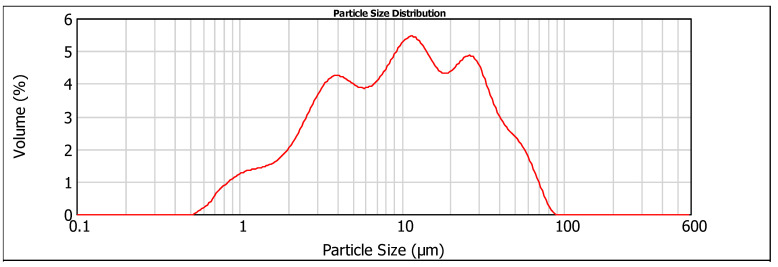
Particle size distribution analysis of CEM IV 32.5.

The laser PSD results (Figure 1 and Figure 2) indicated a similar particle size range for particles below 10 μm, with 50% for both cements. For particles above 10 μm, CEM I 42.5 is finer than CEM IV 32.5, with 90% of the particles below 28.661 μm (Table 4). The apparent specific density of the cement powders was determined according to ASTM C 188-95 [22] using a Le Chatelier flask. Loss on ignition (L.I. %) was measured from room temperature to 1000 °C in an oven.

### 3.2. Influence of Wastewater on Cement Pastes

#### 3.2.1. Heat of Hydration

**Figure 3:**
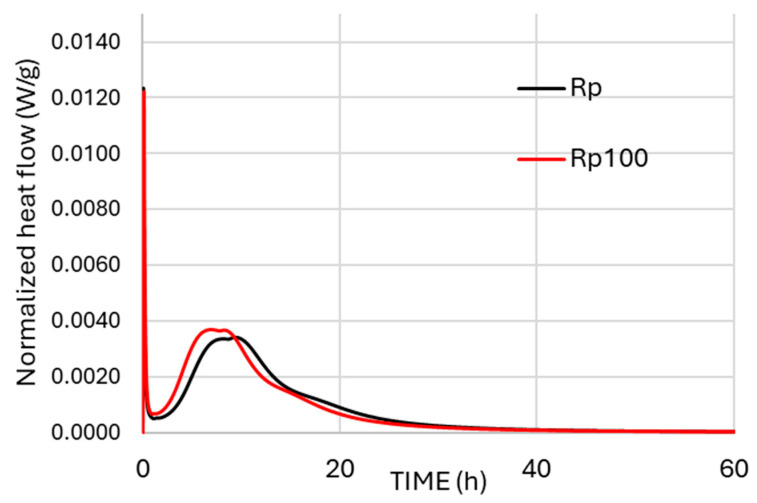
Normalized heat flow (W/g) of CEM I 42.5 with tap water (Rp) and wastewater (Rp100).

**Figure 4:**
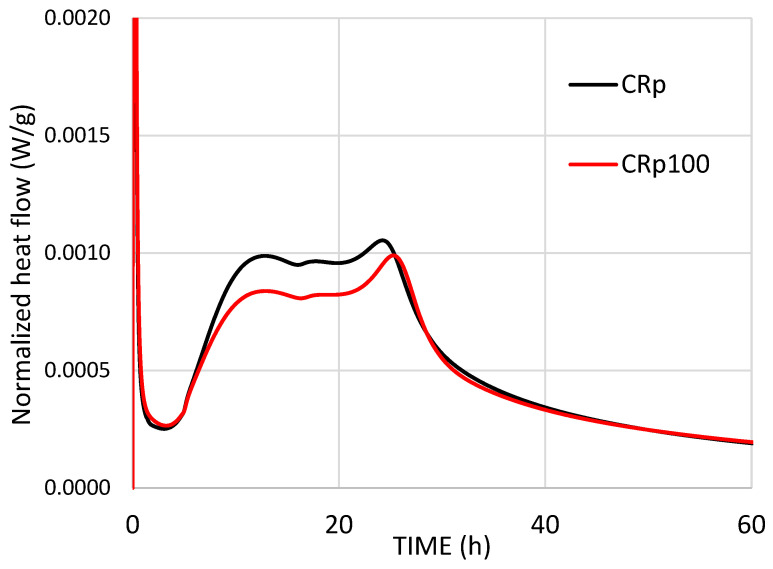
Normalized heat flow (W/g) of CEM IV 32.5 with tap water (CRp) and wastewater (CRp100).

The interaction between cement and water is an exothermic process that releases heat. Figure 3 and Figure 4 show the normalized heat flow of the tested cement pastes during the first 60 h after mixing cement powder with water. In the first stage, a large amount of heat is released, and the initial sharp, narrow peak corresponds to this stage—the immediate heat release. In the second stage, the heat of hydration decreases significantly as cement grains become surrounded by a protective layer of hydration products [33]. When this protective layer collapses, the heat of hydration increases again (third stage) due to further hydration, known as the acceleration period, during which the cement paste sets and hardens. Hydration then continues at a slower rate from this stage onward (fourth stage).

In the case of CEM I 42.5 (Rp and Rp100, Figure 3), replacing tap water with wastewater shifted the time and peak intensities without introducing additional peaks. The addition of wastewater accelerated the third stage and increased the intensity of the corresponding peak. The alkalinity (available OH^−^ anions [34]) and calcium content [8,35] of the wastewater contributed to this acceleration, most likely due to the faster precipitation of portlandite and dissolution of alite.

**Figure 5:**
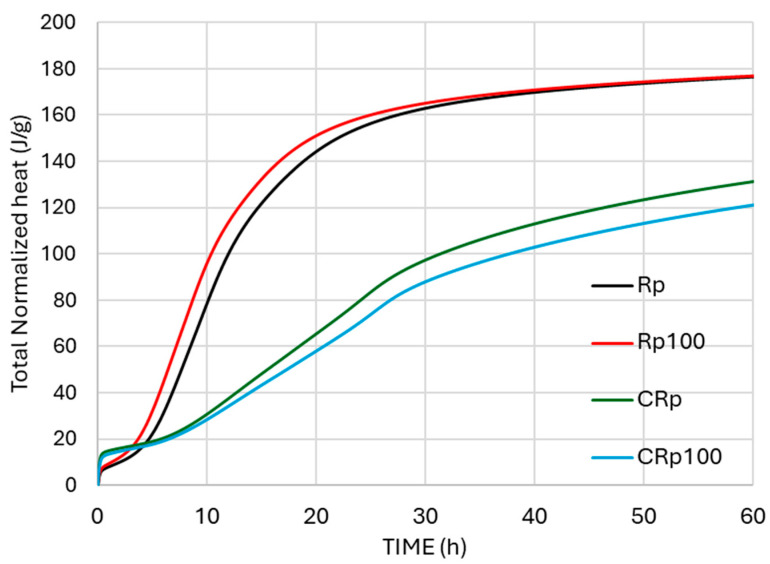
Total normalized heat for the cement pastes with tap water and with 100% replacement with wastewater.

For CEM IV 32.5 (CRp and CRp100, Figure 4), replacing tap water with wastewater shifted only the ettringite formation peak and resulted in lower peak intensities. The total normalized heat was lower when wastewater was used. The last broad peak in Figure 4 is attributed to the supplementary cementitious materials in the blended cement IV 32.5, which are mainly fly ash [36] and natural pozzolan [37]. Fly ash, a fine material, increases peak intensities at the fourth stage of hydration heat measurements [38]. Additionally, the additions of fly ash and natural pozzolan are responsible for the decrease in normalized heat flow and the lower normalized heat (Figure 5) resulting from the hydration of CEM IV 32.5 compared to CEM I 42.5.

Based on the heat of hydration results, the present study demonstrates that the cement type and the presence of supplementary materials in cement affect the hydration kinetics of mixtures with alkaline wastewater, as shown in Figure 3 and Figure 4.

#### 3.2.2. Setting Times

**Figure 6:**
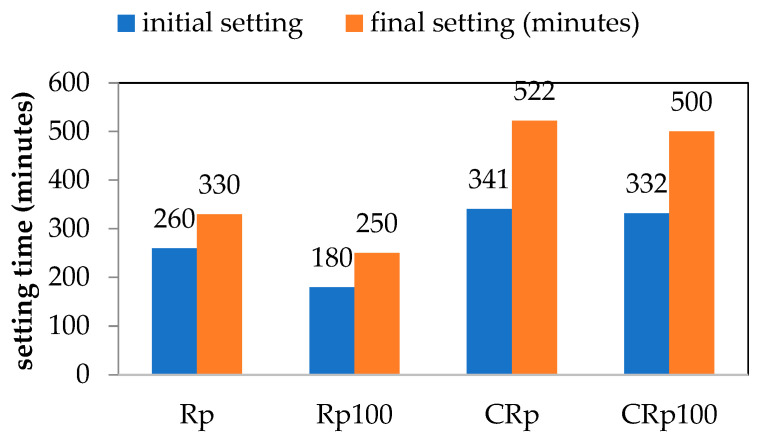
Influence of wastewater on initial and final setting times of cement pastes.

The initial and final setting times for all cement pastes are shown in Figure 6. The addition of wastewater accelerates both the initial and final setting of CEM I 42.5 and CEM IV 32.5. The results for the Rp mixture indicate earlier initial and final setting times, consistent with normalized heat flow recordings (Figure 5), which show accelerated hydration of CEM I 42.5. The setting time of mixtures with wastewater is shorter than that of mixtures with tap water, which aligns with some relevant literature reports [12,16].

Most literature reports mention that setting times increase, usually within acceptable limits, as reviewed in [3]. The type of wastewater is another factor influencing setting time behavior. For instance, Babu and Ramana [39] tested 15 different types of wastewater (all with neutral pH values), and their results indicated that some wastewaters increased setting times while others decreased them. Additionally, the pH level and the presence of metals such as zinc, copper, or iron can prolong setting times. For example, Valipour et al. [15] reported that dissolved salts prolong the setting times of cement. The type of cement also plays an important role. Borger et al. [40] compared OPC type II and type III (ASTM classification) and found that type II cement showed a greater retardation trend compared to type III cement.

#### 3.2.3. Compressive Strength

**Figure 7:**
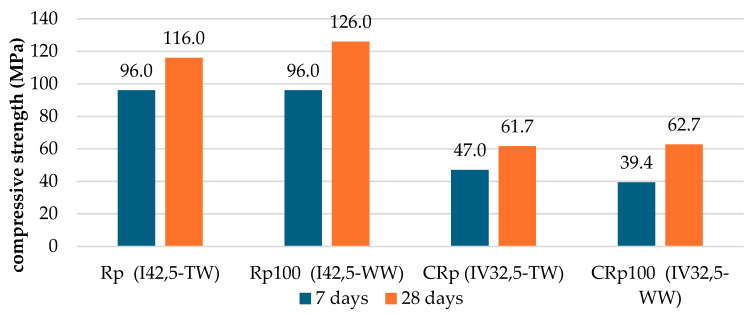
Compressive strength of cement pastes with tap and wastewater at 7 and 28 days (TW stands for tap water and WW stands for wastewater).

Figure 7 compares the compressive strength development of cement pastes made with two different cement types and two water sources (average of three specimens per testing age). CEM I 42.5 was hardly affected by the use of wastewater compared to tap water. At 7 days, the results were equal, and at 28 days, the compressive strength of Rp100 was slightly higher than that of Rp. For CEM IV 32.5, the 7-day results show that replacing tap water with wastewater decreased the compressive strength of CRp100 by almost 20% compared to CRp. Despite this early strength reduction, the 28-day compressive strength was unaffected.

System Rp100 contained OPC powder and showed that clinker can interact with wastewater and that cement hydration progressed normally, as indicated by the heat of hydration and compressive strength results. Early age mechanical properties seem practically unaffected by the wastewater replacement, which contradicts some of the previous research works mentioning a decrease in compressive strength after wastewater replacement in cement pastes.

The cement paste testing showed that wastewater did not interfere with the development of the mechanical characteristics of cement. Chatveera & Lertwattanaruk [12] reported a reduction in compressive strength by 5–6%, determined in cement pastes. Vaičiukynienė et al. [17] reported a slight increase in compressive strength at 7 days with OPC, but an 8.2% reduction at 28 days. However, they also noted no effect at 28 days when concrete washwater was combined with a zeolitic by-product. Unfortunately, in most cases of cement pastes with wastewater, the testing was limited in calorimetry testing or rheology determinations [5,8], without compressive strength results. Accordingly, the literature results for mortar testing are sufficient, but the results for cement pastes are limited.

In the cement pastes tested, the addition of wastewater seemed to improve the mechanical properties of two different types of cement, OPC and blended-type pozzolanic cement. The evolution of compressive strength was slightly enforced in the case of OPC and was slowed down only at the early age in the case of the pozzolanic cement. Indeed, the compressive strength test results of Rp100 in Figure 7 agree with the faster setting times (Figure 6) and the increased heat flow (Figure 4). Additionally, the compressive strength test results of CRp100 are in agreement, with moderate variations, with the setting times and lower heat flow peak intensities (Figure 5).

#### 3.2.4. Thermal Analysis and Thermogravimetry

Table 5 presents the mass loss percentages of the tested cement pastes and the calcium hydroxide content (wt%) (portlandite content, Ca(OH)_2_) calculated from the mass loss in the temperature range of 350 °C to 550 °C. Mass loss between 50 °C and 250 °C indicates the evaporation of non-bonded water and the decomposition of ettringite. Mass loss between 600 °C and 800 °C is due to the decomposition of carbonated compounds.

**Figure 8:**
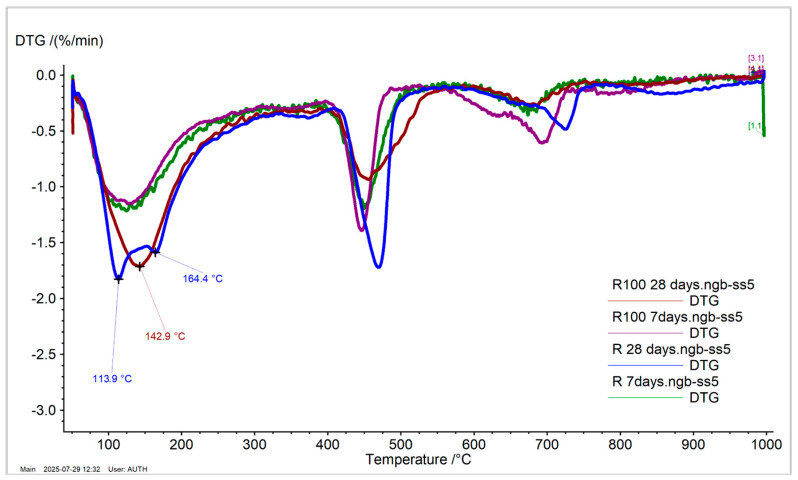
DTG curves of CEM I 42.5 pastes with tap and wastewater at 7 and 28 days, respectively.

At 28 days, the mass loss of Rp between 50 °C and 250 °C, and the calcium hydroxide content (Ca(OH)_2_ wt%), increased compared to the values at 7 days due to the progression of hydration [35], regardless of the type of water added. Figure 8 shows the derivative thermogravimetric (DTG) curves of the Rp systems, where the Rp cement paste exhibits two distinct decompositions at 113.9 °C and 164.4 °C (Rp at 28 days), attributed to non-bonded water and ettringite, respectively. In contrast, Rp100 shows a single broad peak with a maximum at 142.9 °C, likely due to a shift in the decomposition of non-bonded water. Portlandite content increases over time, from 7 to 28 days, consistent with literature findings [41,42]. However, the addition of wastewater leads to a decrease in portlandite content at both tested ages. Additionally, the carbonate species of Rp and Rp100 at 28 days show different peak maxima. The shift in Rp100 to a lower temperature is likely related to differences in carbonate crystallinity [43].

**Figure 9:**
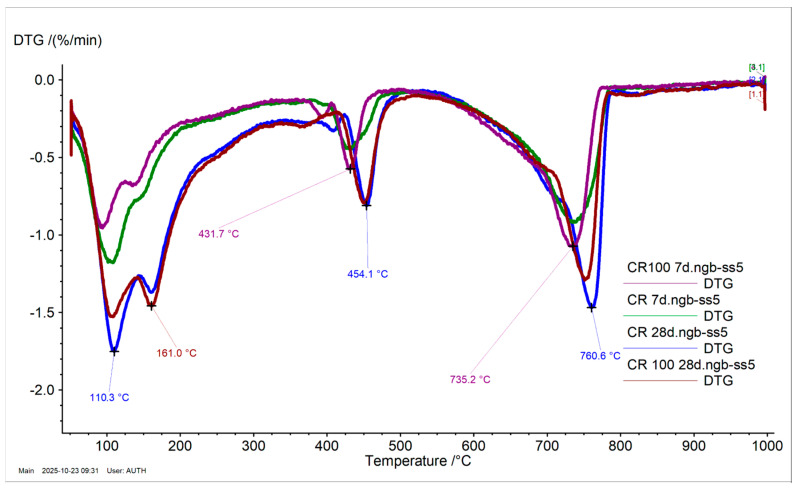
DTG curves of CEM IV 32.5 pastes with tap and wastewater at 7 and 28 days, respectively.

In the CR100 cement paste, the results are similar to those of the CR paste (Figure 9). Therefore, the addition of wastewater did not affect the mass loss results or the calcium hydroxide concentration in the systems. The differences in the thermal analysis curves of cement paste with CEM IV 32.5 compared to CEM I 42.5 are attributed to the different compositions of the cement powders, which reflect different hydration trends, consistent with the hydration kinetics results shown in Figure 3, Figure 4 and Figure 5.

### 3.3. Influence of Wastewater on Concrete

After evaluating wastewater from concrete truck washing in two different types of cement pastes, it was determined that the type of cement significantly influences the compressive strength behavior when wastewater is added. It is likely that clinker can interact with alkaline wastewater, as CEM I 42.5 pastes showed a slight increase in compressive strength, while CEM IV 32.5, which contains large amounts of pozzolanic material, showed a slight decrease in early-age compressive strength. The next step in evaluating wastewater in construction was its application in a concrete mixture to test its mechanical properties and durability.

#### 3.3.1. Fresh Concrete Properties

For the test concrete mixtures, a constant water-to-binder ratio was maintained for comparison purposes. The density of wastewater varied in the second decimal place, and the residual mass was found to be very low (0.2 wt%) according to Table 3. In 176 kg/m^3^, the residual material is calculated to be 0.352 kg/m^3^ or 352 g/m^3^. This means that the water-to-binder ratios of concrete with tap water and wastewater would be comparable.

In concrete mixtures where wastewater replaced tap water, most literature reports a reduction in slump. de Matos et al. [5], Sandrolini and Franzoni [44], and Su et al. [45] observed slump reduction at both 0 min and 60 min after concrete preparation. In contrast, Tsimas and Zervaki [46] reported no change in slump measurements. In the present study, comparison between the control mix and concrete with wastewater showed that using wastewater resulted in increased slump at both 0 min and 60 min after preparation (Table 4), although the rate of slump reduction after 60 min was similar in both mixtures.

#### 3.3.2. Flexural and Compressive Strength

**Figure 10:**
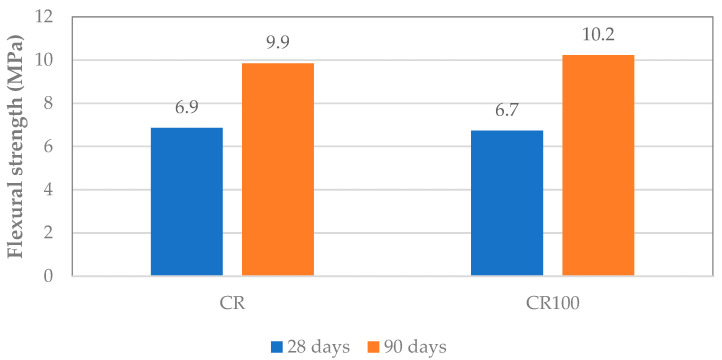
Flexural strength development of CR and CR100 concretes.

**Figure 11:**
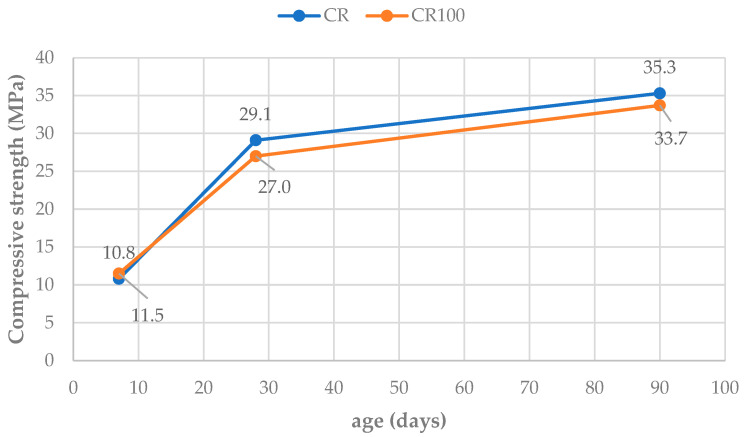
Compressive strength development of CR and CR100 concretes.

Flexural and compressive strengths were determined in prismatic and cubic concrete specimens at 28 and 90 days, and at 3, 28, and 90 days, respectively (average of three specimens per testing age). Flexural strength values shown in Figure 10 indicate a negligible influence of water type on this property. The results are comparable at both testing ages. Additionally, compressive strength results show that replacing tap water with wastewater led to a minimal decrease in compressive strength, with a CR100 decrease rate of less than 3% compared to CR (Figure 11). Practically, the mechanical properties appeared unaffected by the use of wastewater, up to 90 days.

The above results align with literature reports, which indicate that the flexural strength of concrete made with wastewater is similar to that of the control mix [47] or is slightly and insignificantly decreased [48,49]. The literature on compressive strength test results shows either a slight increase, ranging from 4% to 10% in some reports [9,50], or a slight decrease in others [5,16,48,49]. de Matos et al. [5] reported that replacing 50% of water with recycled water from mixer truck wash resulted in 94% of the compressive strength of the reference at 28 days. Additionally, complete replacement of tap water with recycled water achieved 92% of the compressive strength. In the present study, the compressive strength of concrete with wastewater reached 90% of the control concrete values.

#### 3.3.3. Capillary Water Absorption and Permeability

**Figure 12:**
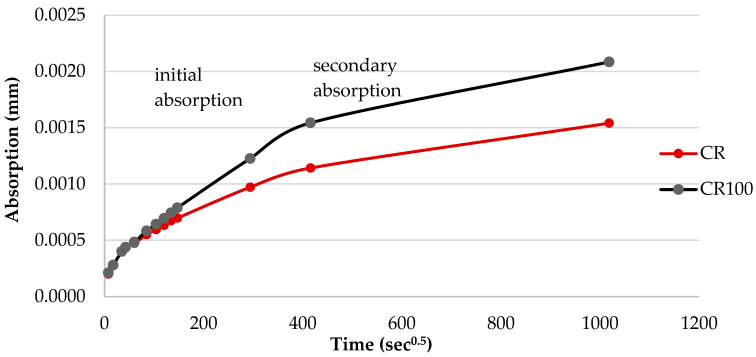
Capillary absorption curve of CR and CR100 concrete samples, at 28 days.

Capillary absorption results, shown in Figure 12, indicate a small increase in water absorption in CR100 specimens, suggesting that more or wider capillary voids formed in CR100 specimens compared to CR. Initial capillary absorption occurs during the first 0 to 6 h of water exposure and reflects the filling of the pore structure with water. In the secondary absorption phase, the rate slows down. Figure 12 demonstrates that 100% replacement of tap water with wastewater leads to a higher absorption rate during the first 6 h, which can be attributed to changes in the pore structure of concrete (open porosity or pore size distribution).

It has been reported that using wastewater in concrete reduces capillary water absorption due to the filling action of fines present in the wastewater, which refines porosity [44]. In the present case, the wastewater characterization showed a residual mass of 0.2 wt% (Table 1), indicating the absence of fines.

**Figure 13:**
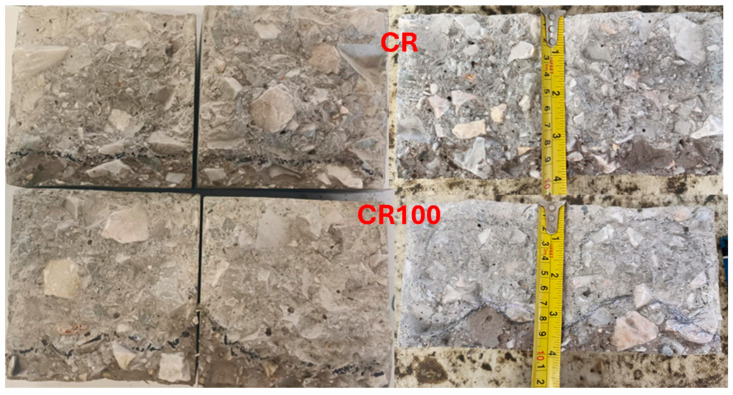
Permeability of representative concrete cubes CR and CR100.

The water penetrability of CR and CR100 concrete specimens did not yield similar results. The penetrability of CR100 showed an uneven penetration depth ranging from 1 mm to 3 mm, while CR exhibited smooth and stable penetration with a depth of 1.5 mm (Figure 13). Nevertheless, when the penetration depth is below 50 mm, concrete is considered impermeable according to some authors [16,51]. Therefore, both concrete mixtures may be considered impermeable, despite their different responses to surface penetration. Repeating the test produced similar results, with smooth and horizontal water penetration in CR and uneven penetration in CR100. This outcome is likely due to differences in the capillary network of the paste between the aggregates or, additionally, to differences in the interfacial transition zone, and requires further investigation.

#### 3.3.4. Carbonation Resistance

**Figure 14:**
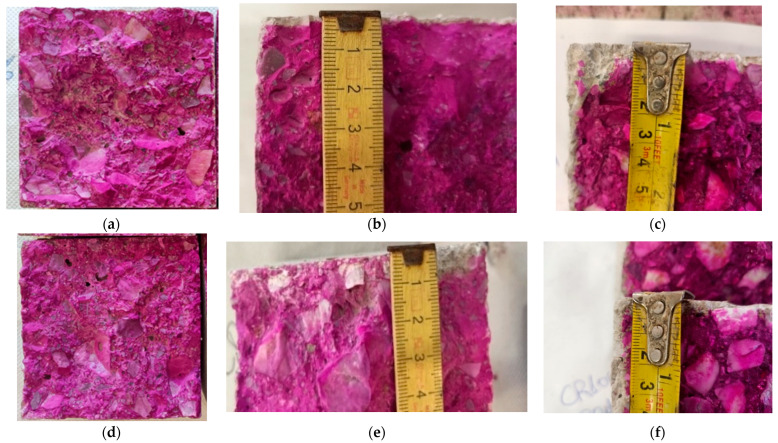
Carbonation resistance of concrete cubes after 7, 28, and 90 days subjected to a CO_2_ 3% chamber. Phenolphthalein testing pictured the carbonated area (gray zone). The purple color shows the alkaline pH of concrete, while discoloration occurs due to pH values smaller than 9.5. (**a**) CR 7 days: Absence of carbonated zone; (**b**) CR 28 days: Mean carbonation depth *d*_k_ = 2.00 mm from the surface; (**c**) CR 70 days: Mean carbonation depth *d*_k_ = 3.00 mm from the surface, uneven on the edges; (**d**) CR100 7 days: Absence of carbonated zone; (**e**) CR100 28 days: Mean carbonation depth *d*_k_ = 2.75 mm; and (**f**) CR100 70 days: Mean carbonation depth *d*_k_ = 3.50 mm.

For the carbonation resistance test, prisms measuring 100 × 100 × 400 mm^3^ were cured in a climatic chamber with 3% CO_2_. After 7, 28, and 70 days of curing, the prisms were broken at 5 cm intervals from their edge, and the inner surface of the concrete section was sprayed with a pH indicator (phenolphthalein solution). The pH indicator changes color based on the pH of the surface; it appears pink in an alkaline environment and becomes colorless at pH values below 9.5. The pH of the carbonated zone of concrete is below 9.5; therefore, the pink areas of the section indicate healthy concrete, while colorless areas indicate carbonated concrete [52]. The corresponding images of the sprayed samples are shown in Figure 14. At 7 days, the concrete specimens resisted carbonation regardless of the water type used. At 28 days, the carbonation zone was found at approximately 2.00 mm depth from the surface. In CR, the carbonation depth was uniform. In contrast, the CR100 concrete had an uneven carbonation zone depth, but the mean carbonation depth (*d*_k_) was 2.75 mm. At 70 days, CR concrete showed a mean carbonation depth of 3.00 mm, which was uneven at the edges, and CR100 concrete showed a 3.50 mm carbonated zone at the surface. Table 6 summarizes the mean carbonation depth values and carbonation rate (mm/√day). Carbonation depth increases over time, and the carbonation rate decreases with the square root of time in all tested concretes. The absence of a carbonation zone at 7 days resulted in zero values, which were excluded from the table.

Carbonation resistance decreased when wastewater was used. The uneven depth of carbonation penetration is attributed to the network of pores in the concrete structure [53], as demonstrated by the variation in carbonation zone thickness, especially in concrete CR100. Using wastewater in concrete as a replacement for tap water increases carbonation depth, consistent with other literature reports [54], but the overall degree of carbonation remains relatively small in both cases.

**Figure 15:**
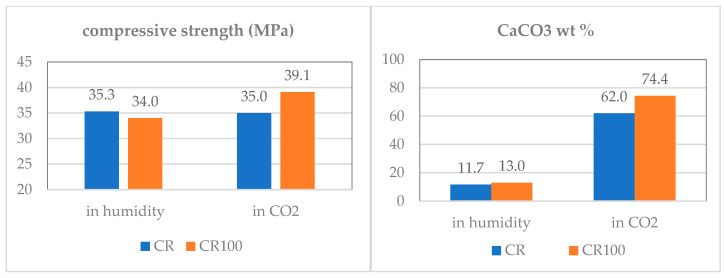
Compressive strength test results (**left**) and calcium carbonate content (**right**) of CR and CR100 concrete cubes, under different curing regimes, after 98 days under humidity and 70 days in a 3% CO_2_ chamber (added to the 28 days of the initial curing in humidity).

After the carbonation test, the concrete specimens were tested in compression, and the compressive strength results are shown in Figure 15. Concrete CR100 cured in CO_2_ showed a 14.70% increase in compressive strength compared to moisture curing and an 11.42% increase compared to CR concrete. Additionally, the calcium carbonate content (Figure 15) from the surface material (1–2 cm depth) showed a 19.35% increase in carbonation when wastewater was used instead of tap water.

It appears that carbon dioxide has penetrated the pores and capillaries of the structure [34,55] and carbonated the cement paste. The increased capillarity contributed to a greater number of available voids in the CR100 specimens and, consequently, to a higher calcium carbonate content compared to the control concrete.

#### 3.3.5. Chloride Resistance

**Figure 16:**
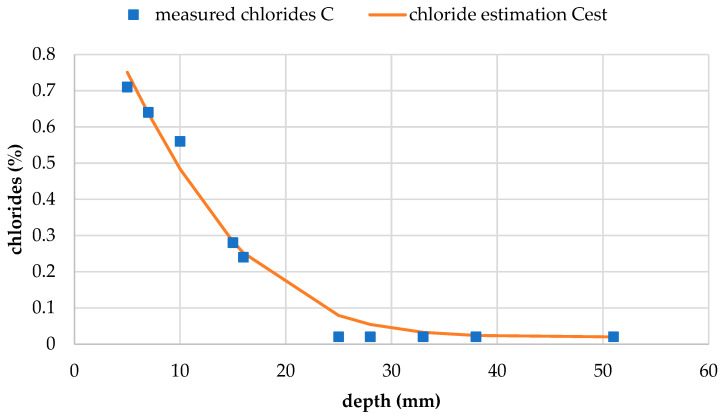
Chloride profile of concrete CR.

**Figure 17:**
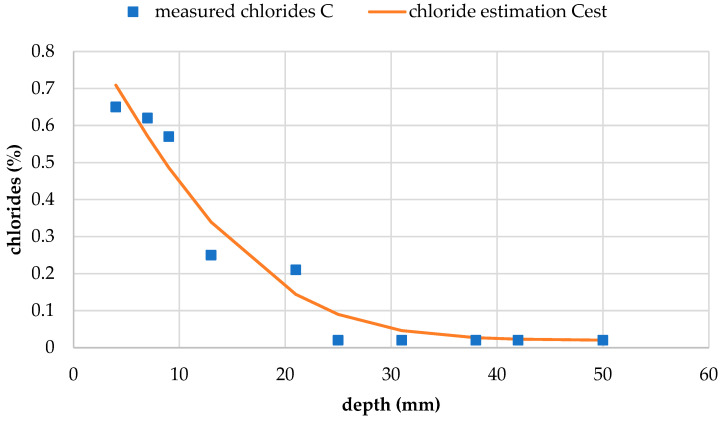
Chloride profile of concrete CR100.

The chloride profiles for test concretes CR and CR100 are shown in Figure 16 and Figure 17, respectively. The initial chloride content, *C*_i_, was measured at 0.02% for both concretes. The calculated chloride content at the exposed surface, *C*_s_, and the non-steady-state chloride diffusion coefficient, *D*_nss_, were determined from the chloride profile curves for each concrete and are presented in Table 7. Although *C*_s_ is higher for reference concrete CR, the diffusion coefficient is higher for concrete with wastewater CR100. This means that despite starting with a higher chloride concentration, chlorides diffuse slightly slower in the reference concrete compared to CR100. This occurrence may be linked with differences in the capillary network of the pores between the two concretes, as for water penetration in Section 3.2.3, and requires further investigation.

**Figure 18:**
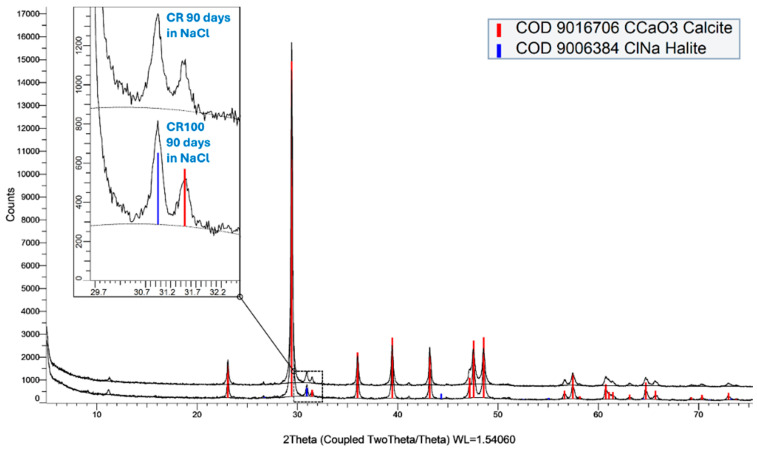
Diffractograms of concrete samples CR and CR100 after 90 days in 3.0 wt% % sodium chloride solution.

Figure 18 shows the form of sodium chloride in the concrete samples (halite, COD 9006384). According to the diffraction patterns of the samples after immersion in 3.0 wt% sodium chloride, the chlorides remain in their original form and have not interacted with the structure of the concrete. The main phase in the red color is calcite (COD 9016706, as given in Figure 18), depicting that at the age of 90 days, the main phase found in concrete is calcite.

The calculated chloride content of the exposed concrete surface shows only minor variation between results, but wastewater concrete exhibits a slightly lower *C*_s_ value and a slightly higher *D*_nss_ value compared to reference concrete. Overall, the chloride resistance of concrete made with wastewater appears comparable to that of the reference concrete. Some literature, such as a study by Hassani et al. [56], reports that the addition of wastewater increases chloride ion penetration. Meena et al. [54] found that the chloride resistance of concrete with tertiary treated wastewater decreased significantly, by almost half compared to the control mix. In both studies, the respective wastewater sources did not introduce alkalinity and had a neutral pH; for example, Hassani reported a pH of 8.1. In the present study, the good chloride resistance of wastewater concrete could be attributed to the alkalinity of the wastewater used.

## 4. Conclusions

The present study showed that adding wastewater does not interfere with the hydration of OPC (CEM I 42.5). It was also concluded that cement powders containing supplementary materials, such as fly ash and natural pozzolan, behave differently during hydration and exhibit decreased hydration kinetics, according to heat flow results, when wastewater is used as a replacement. The compressive strength of CEM I 42.5 cement pastes increased slightly when wastewater completely replaced tap water. In contrast, CEM IV 32.5 cement paste with wastewater showed a decrease in early-age strength compared to the control paste with tap water.

In concrete testing, the addition of wastewater increased water penetrability and slightly decreased carbonation resistance and chloride resistance of concrete, which was interpreted as the effect of wastewater use on the pore structure of the concrete. The combination of wastewater characteristics and its alkalinity, along with the constituents of cement, appears to play a crucial role in the behavior of the concrete mixture regarding the hydration process, capillary structure, and mechanical performance.

## Figures and Tables

**Table 1:** Proportioning of the cement pastes and Vicat consistence.

Paste	CEM IV 32.5 (g)	CEM I 42.5 (g)	Wastewater (g)	Tap Water (g)	Vicat Consistence (mm)
Rp	1000	-	-	540	4
Rp100	1000	-	540	-	5
CRp	-	1000	-	380	4
CRp100	-	1000	380	-	7

**Table 2:** Concrete mix design and fresh concrete properties.

Constituents/Property	CR	CR100
CEM IV 32.5 (Kg/m^3^)	320	320
Tap Water (Kg/m^3^)	176	-
Wastewater (Kg/m^3^)	-	176
Aggregates 0–4 mm (Kg/m^3^)	767	767
Aggregates 4–8 mm (Kg/m^3^)	281	281
Aggregates 8–16 mm (Kg/m^3^)	412	412
Aggregates 16–31.5 mm (Kg/m^3^)	412	412
0’ slump (mm)	30	48
60’ slump (mm)	4	10
Apparent Specific Density (Kg/m^3^)	2383	2370

**Table 3:** Characterization of wastewater based on the requirements of EN 1008:2002 [20].

Property	Measured Values	Limits According to ΕΝ 1008
Residual mass	0.2 wt%	<1 wt%
Cl^−^	16 mg/L	<4500 mg/L
NO_3_^−^	70.4 mg/L	<500 mg/L
SO_4_^2−^	149.6 mg/L	<2000 mg/L
pH	12.86	>4.0
density	1.004 mg/L	<1.02 kg/L
Na_2_Oeq	48.17 mg/L	<1500 mg/L

**Table 4:** Chemical and physical properties of the cement powders used.

Property	Method	CEM I 42.5	CEM IV 32.5
Density (Kg/m^3^)	ASTM C188-95	3135	2903
L.O.I. (%)	1000 °C	4.12	8.92
Cl^−^ (%)	IC	<0.01	0.11
SO_4_^2−^ (%)	IC	2.03	2.42
CaO (%)	XRF	61.7	39.60
MgO (%)	XRF	1.40	0.94
SO_3_ (%)	XRF	2.94	2.68
Fe_2_O_3_ (%)	XRF	3.70	3.20
Al_2_O_3_ (%)	XRF	3.45	5.96
SiO_2_ (%)	XRF	16.1	26.40
K_2_O (%)	XRF	1.30	2.22
Na_2_O (%)	XRF	0.43	0.84
TiO_2_ (%)	XRF	0.24	0.30
Particle size			
d(0.1) (μm)	Laser PSD	2.438	2.098
d(0.5) (μm)	Laser PSD	10.248	10.086
d(0.9) (μm)	Laser PSD	28.661	36.924

**Table 5:** Mass loss (%) determined from TG curves on specific temperature ranges.

Cement Paste	Test Age (Days)	50–250 °C	Mass Loss (%) 350–550 °C	600–800 °C	Ca(OH)_2_ (%)
Rp	7	6.39	4.46	1.54	18.33
Rp	28	8.66	5.74	2.30	23.59
Rp100	7	6.00	3.99	3.16	16.40
Rp100	28	8.22	5.00	1.49	20.55
CRp	7	4.91	1.84	4.78	7.56
CRp	28	7.93	2.77	6.19	11.38
CRp100	7	3.97	1.75	4.97	7.19
CRp100	28	7.90	2.86	5.38	11.75

**Table 6:** Mean carbonation depth and carbonation rate.

Concrete	Mean Carbonation Depth *d*_k_ (mm)		Carbonation Rate (mm/√Days)	
	28 Days	70 Days	28 Days	70 Days
CR	2.00	3.00	0.38	0.36
CR100	2.75	3.50	0.52	0.42

**Table 7:** Calculated chloride content at the exposed concrete surface (*C*_s_) and non-steady state diffusion coefficient (*D*_nss_) for the test concretes.

Test Concrete	*C*_s_ (%)	*D*_nss_ (m^2^s^−1^)
CR	1.059	1.11 × 10^−11^
CR100	0.905	1.30 × 10^−11^

## Data Availability

The original contributions presented in this study are included in the article. Further inquiries can be directed to the corresponding author.

## Abbreviations

The following abbreviations are used in this manuscript:

OPC	Ordinary Portland Cement
EN	European Standard
ASTM	American Society for Testing and Materials
XRF	X-ray fluorescence
IC	Ion chromatography
PSD	Particle size distribution
LOI	Loss on ignition
DTG	Derivative thermogravimetry
